# Role of Exonic Variation in Chemokine Receptor Genes on AIDS: *CCRL2 F167Y* Association with Pneumocystis Pneumonia

**DOI:** 10.1371/journal.pgen.1002328

**Published:** 2011-10-27

**Authors:** Ping An, Rongling Li, Ji Ming Wang, Teizo Yoshimura, Munehisa Takahashi, Ram Samudralal, Stephen J. O'Brien, John Phair, James J. Goedert, Gregory D. Kirk, Jennifer L. Troyer, Efe Sezgin, Susan P. Buchbinder, Sharyne Donfield, George W. Nelson, Cheryl A. Winkler

**Affiliations:** 1 Basic Research Laboratory, SAIC–Frederick, National Cancer Institute–Frederick, Frederick, Maryland, United States of America; 2 Office of Population Genomics, National Human Genome Research Institute, Bethesda, Maryland, United States of America; 3 Laboratory of Molecular Immunoregulation, National Cancer Institute–Frederick, Frederick, Maryland, United States of America; 4 Department of Microbiology, University of Washington School of Medicine, Seattle, Washington, United States of America; 5 Laboratory of Genomic Diversity, National Cancer Institute–Frederick, Frederick, Maryland, United States of America; 6 Division of Infectious Diseases, Feinberg School of Medicine, Northwestern University Medical School, Chicago, Illinois, United States of America; 7 Infections and Immunoepidemiology Branch, National Cancer Institute, Bethesda, Maryland, United States of America; 8 Department of Epidemiology, Johns Hopkins Bloomberg School of Public Health, Baltimore, Maryland, United States of America; 9 BSP/CCR Genetics Core, SAIC–Frederick, National Cancer Institute–Frederick, Frederick, Maryland, United States of America; 10 San Francisco Department of Public Health, San Francisco, California, United States of America; 11 Rho, Chapel Hill, North Carolina, United States of America; Stanford University School of Medicine, United States of America

## Abstract

Chromosome 3p21–22 harbors two clusters of chemokine receptor genes, several of which serve as major or minor coreceptors of HIV-1. Although the genetic association of *CCR5* and *CCR2* variants with HIV-1 pathogenesis is well known, the role of variation in other nearby chemokine receptor genes remain unresolved. We genotyped exonic single nucleotide polymorphisms (SNPs) in chemokine receptor genes: *CCR3*, *CCRL2*, and *CXCR*6 (at 3p21) and *CCR8* and *CX3CR1* (at 3p22), the majority of which were non-synonymous. The individual SNPs were tested for their effects on disease progression and outcomes in five treatment-naïve HIV-1/AIDS natural history cohorts. In addition to the known *CCR5* and *CCR2* associations, significant associations were identified for *CCR3*, *CCR8*, and *CCRL2* on progression to AIDS. A multivariate survival analysis pointed to a previously undetected association of a non-conservative amino acid change F167Y in *CCRL2* with AIDS progression: 167F is associated with accelerated progression to AIDS (RH = 1.90, *P* = 0.002, corrected). Further analysis indicated that *CCRL2*-167F was specifically associated with more rapid development of pneumocystis pneumonia (PCP) (RH = 2.84, 95% CI 1.28–6.31) among four major AIDS–defining conditions. Considering the newly defined role of CCRL2 in lung dendritic cell trafficking, this atypical chemokine receptor may affect PCP through immune regulation and inducing inflammation.

## Author Summary

Human chemokine receptors are cell surface proteins that may be utilized by HIV-1 for entry into host cells. DNA variation in the HIV-1 major coreceptor CCR5 affects HIV-1 infection and progression. This study comprehensively assesses the role of genetic variation of multiple chemokine receptor genes clustered in the chromosome 3p21 and 3p22 on HIV-1 disease outcomes in HIV-1 natural history cohorts. The multivariate survival analyses identified functional variants that altered disease progression rate in *CCRL2*, *CCR3*, and *CCR8*. *CCRL2*-F167Y affects the rate to AIDS development through a specific protection against pneumocystis pneumonia (PCP), a common AIDS–defining condition. Our study identified this atypical chemokine receptor CCRL2 as a key factor involved in PCP, possibly through inducing inflammation in the lung.

## Introduction

Functional variation in the human leukocyte antigen (*HLA*) class I genes and in chemokine receptors affects HIV susceptibility, viral load, and rates of disease progression [1]–[4]. Recent genome-wide association studies (GWAS) performed in HIV-1 cohorts have shown that the *HLA* region and the chemokine receptor *CCR5* gene have major roles in control of HIV-1 replication and disease progression—together they explain approximately 20% of genetic variability [3], [5]–[8] (reviewed in [4], [5]). These findings from GWAS highlighted the leading role of chemokine receptors among non-*HLA* genes in HIV-1 pathogenesis and prompted us to assess the role of other chemokine receptor genes on HIV disease using a gene-centric approach to identify common or rare functional variants in the region.

The chemokine receptor cluster on chromosome 3 contains at least 12 genes including *CCR5*, the primary HIV-1 co-receptor [9]–[11]. Multiple genetic variants in chemokine receptors and chemokines have been identified as modifiers of HIV-1 infection or disease progression [12], [13], including *CCR5*-Δ32 (a 32-bp deletion introduces a premature stop codon) [14] and *CCR5* promoter variants [15]–[17] and variants in the CCR5 ligand gene *CCL5* [18], [19]. The homozygous *CCR5* Δ32/Δ32 genotype and complex heterozygotes with other rare amino acid mutations confers near complete resistance to HIV infection [12], [14], [20]–[22]. Individuals homozygous for a haplotype known as *CCR5*-P1 [15] or haplogroup HHE [23], a multisite allele of the *CCR5* promoter region, progress to AIDS more rapidly than those with other *CCR5* promoter haplotypes [15]–[17], [23]–[25]. CCR2 and CXCR6 are minor HIV-1 coreceptors used by a limited number of HIV-1 strains as an entry coreceptor [26], [27]. *CCR2*-V*64I* has been associated with delayed progression [12], [25], [28], [29]. Variants in *CXCR6* were also associated with disease modification [30], [31].

**Figure 1:**
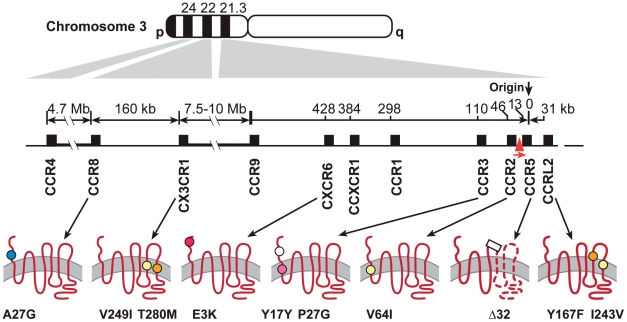
Polymorphisms in seven chemokine receptor genes on the Chromosome 3p21–22. Schematics at bottom show the configuration of the receptors in the cell membrane, with circles representing the position and type of the polymorphisms. White circle indicates a synonymous substitution, colors nonsynonymous substitutions: yellow, conservative; blue, hydrophobic—hydrophilic; orange, hydrophilic—hydrophobic; red, charge changing (acidic—basic), purple, shape changing. Also indicated are Δ32 in *CCR5* (rectangle) and the polymorphic *CCR5* promoter (red triangle in the gene map). *CCR5*-2459 promoter allele (rs1799987).

The chromosome 3 chemokine receptor cluster extends from 3p21 to 3p24, with eight receptors occurring in an 520 kb region of 3p21(Figure 1) [32]. The cluster contains genes for several receptors, CCR3, CCR8, CX3CR1, and CXCR6 that have been shown to bind HIV env or to support varying levels of in vitro replication of HIV-1, HIV-2 or simian immunodeficiency virus (SIV) [26], [33]–[36] (reviewed by [11], [37]). The role played by minor coreceptors in HIV-1 pathogenesis is not clear, but studies have suggested that a broad spectrum of coreceptor usage may be correlated with rapid CD4+ cell depletion and AIDS progression [11], [38], [39]. Primary isolates of HIV-1 have been shown to use a wide spectrum of various chemokine receptors as HIV coreceptors [40]. HIV-1 isolates from a *CCR5*-Δ32 heterozygous or homozygous individual can use various minor coreceptors such as CCR3, CCR2B, CCR8, CX3CR1 for cell entry [41], [42]; amino acid mutations in the V3 loop of HIV-1 are responsible for utilization of multiple coreceptors [40]. Considering the high mutation rate and sequence heterogeneity of HIV-1, particularly within the *env* gene, it is plausible that a spectrum of receptors is used *in vivo* during the course of HIV infection and that genetic variants in the coreceptors may affect usage or binding efficiency by HIV-1. Furthermore, as CCR5 and CXCR4 antagonists blocking these major co-receptors are used therapeutically [37], the potential of HIV-1 to evolve to use other minor coreceptors as alternative cell entry points is expected to increase. Therefore, determining whether HIV-1 minor coreceptor genes, in addition to CCR5 and CXCR4 play a role in HIV pathogenesis is a timely topic.

In this study, we evaluate the impact of chemokine coreceptors (CCR) on HIV/AIDS using a candidate-gene based population association analysis in five treatment-naive HIV-1 natural history cohorts. Genotypes of exonic polymorphisms in *chemokine coreceptor genes CCR3*, *CCRL2* and *CXCR6* on Chromosome 3p21 and in *CCR8* and *CX3CR1* on 3p22 were tested for their genetic influence on AIDS progression. CCR3, CCR8 and CXCR6 were chosen as they are HIV-1 minor coreceptors [26], [33]–[36] (reviewed by [11], [37]). CCRL2 was selected as a candidate gene because of its homology with CCR5 (45%)—the most of any of the chemokine receptors genes—because of its proximity to CCR5, and because it is an atypical receptor without signal transduction, similar to DARC. Our results suggest that genetic variation in *CCR3*, *CCR8* and *CCRL2* may contribute additional genetic regulation of HIV-1 disease in addition to that conferred by the major HIV-1 coreceptor gene *CCR5*.

## Results

### Identification of chemokine coreceptor variants

We resequenced selected chemokine receptor genes in the chromosome 3p21–22 region in 72 African Americans and 72 European Americans with three extreme phenotypes (resistance to HIV infection, very rapid or slow progression to AIDS) to assess the extent of exonic variation and to identify rare variants. We observed a total of 6 exonic variants, 5 of which were nonsynonymous, in *CCR3*, *CCR8*, *CXCR6*, and *CCRL2* (Figure 1, Table 1). We did not resequence *CCR5* and *CCR2* since these receptor genes had been previously sequenced in HIV patients [28], [43], nor did we resequence *CX3CR1*. We previously showed that *CX3CR1*-V249I and -T280M had no effect on AIDS progression in our group of seroconverter subjects [44], and therefore did not include them in this analysis. No noticeable differences in SNP frequency among three extreme phenotype groups were observed (data not shown). These SNPs were then genotyped in 5 HIV-1 natural cohorts comprising 2594 European Americans (EA). All SNPs were in Hardy-Weinberg Equilibrium (*P*>0.05). *CXCR6*-E3K and *CCR3*-P39L were rare (<1%) in EA and were excluded from the analysis; other SNPs were common (>5%) (Table 1).

**Table 1:** Characteristics and allele frequencies of the chemokine receptor variants.

Gene	Variant	dbSNP ID	Chromosome Position^1^	Domain^2^	EA freq.^3^	AA freq.^3^
***CX3CR1***	*CX3CR1*-V249I	rs3732379	39282260	TM6	0.261	0.140
***CX3CR1***	*CX3CR1*-T280M	rs3732378	39282166	TM7	0.159	0.040
***CCR8***	*CCR8*-A27G	rs2853699	39348906	N-terminus	0.306	0.144
***CXCR6***	*CXCR6*-E3K	rs2234355	45962984	N-terminus	0.006	0.438
***CCR3***	*CCR3*-255C (Y17Y)^4^	rs4987053	46281704	N-terminus	0.083	0.103
***CCR3***	*CCR3-*P39L	rs5742906	46281769	TM 1	0.004	0.013
**CCR2**	CCR2-V64I	rs1799864	46374212	TM 1	0.1	0.15
**CCR5**	*CCR5*-2459A^5^	rs1799987	46386939	promoter	0.56	0.42
**CCR5**	*CCR5*-Δ32	rs333	46389951	Intracellular	0.1	0.01
***CCRL2***	*CCRL2*-Y167F	rs3204849	46425074	3rd ECL	0.394	0.134
***CCRL2***	*CCRL2*-I243V	rs3204850	46425301	TM 6	0.085	0.016

1 Chromosome position is based on the NCBI genome build 36.3;

2 TM, Transmembrane domain; ECL, extracellular loop;

3 Variant allele frequency (freq.) was obtained from participants in AIDS cohorts consisting of European American (EA) and African American (AA);

4 Synonymous nucleotide T/C change at position 255 in the GenBank sequence U51241;

5 Nucleotide G/A change at 2459 bp upstream of *CCR5* ORF.

### Linkage disequilibrium (LD) of chemokine receptor variants on 3p21–22

The SNPs considered fell into two distinct haplotype blocks (Figure 1). The larger block in 3p21 comprises *CCRL2*-F167Y, *CCRL2*-I243V, *CCR5*-+/Δ32, the *CCR5*-promoter SNP *CCR5*-2459A (rs1799864, previously shown to affect CCR5 expression levels and modify AIDS progression [15], [16]), *CCR2*-V64I, *CCR3*-P39L and *CCR3*-255T/C (Y17Y), spanning 77 Kb, and *CXCR6*-E3K, 318 Kb teleomeric to *CCR3*. The second block in 3p22, ∼10 Mb from the first block, consists of *CX3CR1*-V249I and -T280M and *CCR8*-A27G (rs2853699), which are separated by ∼160 Kb. There is moderate to substantial LD within the closely spaced *CCR3*-*CCR2*-*CCR5*-*CCRL2* group (with pairwise D′ 0.47–1, Figure S1). In the 3p22 block, low levels of LD were observed between *CCR8* and *CX3CR1* SNPs (D′ ∼0.40). The LD level between 3p21 and 3p22 blocks is minimal.

### Genetic associations with progression to AIDS

**Figure 2:**
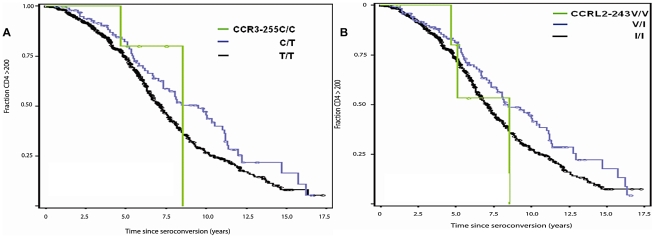
Genetic effect of *CCR3*-255C and *CCRL2*-243V on AIDS progression. Kaplan-Meier survival curves of time progression since HIV-1 infection to AIDS (1987 CDC definition) were plotted for genotypes of *CCR3*-255C (A) and *CCRL2*-243V (B). Circles on the curves indicate those censored.

The identification of independent additional genetic factors in this region, particularly for 3p21, is complicated by a moderate to high level of LD (Figure S1). To detect genes or markers additional to a primary predisposing variant(s) in a genetic region of high LD, stratification analyses or using a restricted dataset are frequently employed [45]. Stepwise regression is also a choice for assessing the relative importance of different variants in the linked region [46]. These approaches are feasible as the SNPs in this region have a low to moderate correlation (for r^2^, see Figure 2).

### Effects of individual variants on AIDS progression

**Figure 3:**
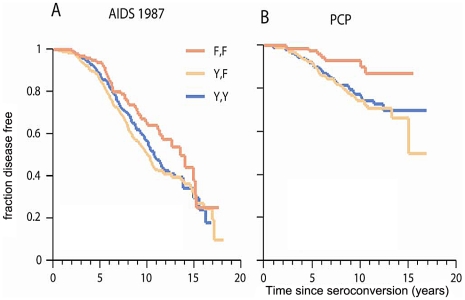
Genetic effect of *CCRL2-*167F on AIDS progression and PCP. Kaplan-Meier survival curves of time progression since HIV-1 infection to AIDS (1987 CDC definition) (A) or to PCP (B) were plotted for *CCRL2-*Y167F genotypes. Circles on the curves indicate those censored.

SNPs with allele frequencies of at least 5% were analyzed by Kaplan-Meier survival curve analysis and Cox proportional hazards model. We present the relative hazards (RH) of the individual variants on survival to AIDS from analysis of 670 EA seroconverters using multivariable Cox regression models in Table 2. Four of the five SNPs in the 3p21 block revealed new significant associations with delayed time progressing to AIDS, after conditioning on *HLA* alleles and *CCR5*-Δ32 (Table 2). Significant associations with differential progression to clinical AIDS were observed for *CCR3* -255C (RH = 0.62, *P* = 0.009, Figure 2A), for *CCRL2*-243V (RH = 0.66, *P* = 0.03, Figure 2B), and for *CCRL*2-167F (RH = 1.89, *P* = 0.0003, Figure 3A) and for *CCR8*-27G (RH = 1.44, *P* = 0.004) (Table 2). Adjusting for potential population stratification had little affect on the significance of the associations (Table 2). Correcting for 7 chemokine receptor variants tested using a Bonferroni step-down (Holm) correction [47], the associations for *CCRL*2-167Y, *CCRL2*-243V, and *CCR*3-255C remained significant (*P* = 0.002, 0.03, and 0.016, respectively), in addition to *CCR5*-Δ32 and *CCR5*-2459, while *CCR8*-27G became non-significant (Table 2).

**Table 2:** Effects of chemokine receptor variants on AIDS progression.

		*Adjusted by HLA alleles* 1			*Adjusted for population stratification* 2			
Variant	Model	RH	95% CI	*p_raw_*	RH	95% CI	*P_eigen_*	*P_Holm_* 3
***CCR3*** **-255C**	dom	0.62	0.43–0.89	0.009	0.59	0.40–0.85	0.004	0.016
***CCR2*** **-64I**	dom	0.78	0.58–1.05	0.10	0.79	0.57–1.09	0.14	0.14
***CCR5***- 2459A	rec^4^	1.51	1.18–1.93	0.001	1.66	1.27–2.15	0.0002	0.001
***CCR5*** **-Δ32** 5	dom	0.63	0.46–0.86	0.003	0.58	0.42–0.80	0.0008	0.004
***CCRL2*** **-F167**	dom	1.89	1.34–2.66	0.0003	1.90	1.33–2.71	0.0004	0.002
***CCRL2*** **-243V**	dom	0.66	0.46–0.96	0.03	0.60	0.40–0.89	0.01	0.03
***CCR8*** **-27G**	dom	1.44	1.13–1.85	0.004	1.34	1.03–1.73	0.028	0.056

1 The rate of progression to AIDS (1987 CDC definition [66]) were calculated with the Cox proportional hazards model. The analyses were adjusted for age, sex and covariates *CCR5*-Δ32, and four *HLA* alleles (Class I homozygosity, B27, B35Px and B57). CI (confidence interval), RH (relative hazard);

2 After including eigenvector values for the top two principle components as a covariate in the Cox regression analysis to control for potential population stratification. Eigenvector values were obtained from Eigensoft program by performing a principal component analysis of 700,022 SNPs in the previous GWAS study [71];

3 The adjusted *p*-values further correcting for multiple testing of 7 chemokine receptor variants using the Holm's step-down Bonferroni method;

4 *CCR5*-2459A (rs1799987, Tag SNP of *CCR5*-P1, previously shown to be associated with AIDS in a recessive model [15];

5 With adjustment of *CCR5*-2459A.

### Effects of combined chemokine coreceptor variants on AIDS progression

Using stepwise selection determined by the Akaike information criteria (AIC), we built an optimal prediction model with the genetic variants that were associated with AIDS progression in this or previous studies (Table 3). From Table 3, we can see the minimum AIC is achieved at the ninth step with nine covariates. Therefore, the best predictive model accounting for the rate of progression to AIDS (1987 CDC definition) includes the newly identified variants *CCRL2*-167F and -243V, *CCR8*-27G, as well as previously known variants: the two locus *CCR2*-V64I-*CCR5*-Δ32 composite genotype, *CCR5*-2459, *HLA* class 1 homozygosity, HLA-B*35Px, HLA-B*57 and HLA B*27 (without covariates, AIC:1796.32, with 9 covariates, AIC:1737.28). The significance level and RH values in this model represent that of each variant after considering all other linked and unlinked variants (Table 3). The rough shrinkage estimate is 0.88 (104/140), indicating that the model is fairly reliable without further shrinkage or data reduction treatment [48]. We calculated that R^2^ = 0.11 for the final fitted model, indicating that 11% of the variability in AIDS progression can be explained with the variants in the model.

**Table 3:** Sequential stepwise regression for Cox PH model selection and maximum likelihood estimates of genetic covariates associated with AIDS progression.

	Variable	AIC with Covariates^1^	The final model with 9 covariates^3^ (AIC = 1737.28)		
			Estimate/S.E.	RH (95% CI)	*P*
**1**	*HLA B35* px dom	1783.70	0.86/0.0.23	2.37(1.52–3.69)	0.0001
**2**	*HLA B27* dom	1770.23	−1.06/0.31	0.35(0.19–0.64)	0.0007
**3**	*HLA* homozygosity	1766.52	0.31/0.12	1.36 (1.08–1.71)	0.009
**4**	*CCRL2*-243V dom	1761.29	−0.64/0.24	0.53 (0.33–0.84)	0.007
**5**	*CCRL2*-167F dom	1753.95	0.66/0.23	1.93 (1.23–3.01)	0.004
**6**	*CCR5*-Δ32 or *CCR2*-64I	1747.70	−0.54/0.17	0.58 (0.42–0.82)	0.002
**7**	*CCR8*-27G dom	1742.41	0.43/0.15	1.53 (1.15–2.04)	0.004
**8**	*B57* dom	1737.32	−0.80/0.37	0.45 (0.22–0.92)	0.028
**9**	*CCR5*-2459A rec	**1737.28** 2	0.26/0.18	1.30 (0.91–1.87)	0.15
**10**	*CCR3*-255C dom	1738.21			

NOTE. AIC, Akaike information criteria; S.E., standard error; RH, relative hazard; CI, confidence interval; dom, dominant genetic model; rec, recessive model.

1 AIC without covariates (null model) = 1796.32;

2 The best model with minimal AIC is achieved at the ninth step including first 9 covariates entered.

3 The *X* ^2^ value for likelihood ratio test, degree of freedom, 9; *P* = 6.24×10^−13^; and shrinkage of the model = (77.04−9)/77.04 = 0.88. All analyses were stratified with age groups that known to modify AIDS progression.

### *CCRL2*- Y167F is associated with progression to AIDS

The strongest new association was for *CCRL*2-Y167F with progression to clinical AIDS. The common 167F allele (allele frequency 67% in EA) had a significant dominant detrimental effect on progression to AIDS-defining conditions (CDC 1987 case definition) (RH = 1.89, *P* = 0.0003, Wald test; *P* = 0.0001, Likelihood ratio test; n = 670 seroconverters) in the Cox proportional hazards model (Table 2 and Table 3, Figure 3A). When the analysis was restricted to individuals not carrying *CCR5*-Δ32 (n = 539), the association of *CCRL2-*167F with AIDS remained significant (RH = 1.69, 95% CI 1.20–2.38), indicating that the *CCRL2*-167F association is independent of *CCR5*-Δ32 and not due to its linkage disequilibrium with the latter.

### *CCRL2*- Y167F and pneumocystis pneumonia (PCP)

 In an explanatory analysis, we tested whether *CCRL2*- Y167F affects AIDS progression through a specific AIDS-defining illness. A Cox model analysis in the seroconverter group showed significantly faster progression to PCP for carriers of 167F (RH = 2.84, 95% CI 1.28–6.31, *P* = 0.007; Figure 3B), but no differential effect on Kaposi's sarcoma, microbacterial avium infection, cytomegalovirus, or lymphoma (*P*>0.05, data not shown).

To assess whether the *CCRL2*-167F association with PCP was due to LD (D′ = 0.76, r^2^ = 0.47) with *CCR5-*2459 that associated with AIDS progression [16], we restricted the analysis in a group of SC (n = 365) that do not carry the homozygous *CCR5*-2459 genotype; the *CCRL2*-167F association with PCP remained significant (OR = 2.42, 95% CI 1.09–5.36), in support of its independent role.

### Possible function of *CCRL2* F167Y

#### Evolutionary conservation of *CCRL2*

We assessed the homology of *CCRL2* with other C-C chemokine receptor family genes. *CCRL2* shares over 40% sequence homology with known chemokine receptors such as CCR1, -2, -3 and -5 but lacks the DRYLAIV motif in the second extracellular loop that is usually required for the signaling capacity of chemokine receptors [49]. The alignment of mammalian CC chemokine coreceptor sequences shows that the 167 site and surrounding region are highly conserved (Figure S2). Phenylalanine (F) at position 167 is apparently ancestral as it occurs in chimpanzee and macaque. Phenylalanine (F) or other nonpolar amino acids occur at the position homologous to human *CCRL2-167* in 78 of the 82 chemokine receptors, including all receptors known to be functional, while a polar amino acid occurs at this position in two cases for the non-signaling *FY* DARC receptor. Tyrosine (Y) never occurs at this position except for human *CCRL2*. These observations suggest that the F167Y change might have functional consequence.

#### Impact of F167Y on CCRL2 topology

The impact of F167Y on CCRL2 transmembrane (TM) topology was assessed using ConPred II, [50]. 167F is located in the third extracellular loop adjacent to the 4^th^ TM domain. The F to Y substitution introduces a hydrophilic residue that changes the boundary of the TM domain and the conformation of the nearby domains: TM domains 3, 4 and 5 were formed by residues 104–124, 146–166 and 201–221 with CCRL2 carrying 167F, but were formed by residues 103–123, 145–165 and 202–222 with CCRL2-167Y, respectively (Figure S3).

#### Three-dimensional protein structure prediction

The effect of the Y167F amino acid substitution on the structure of the CCRL2 receptor was predicted by PROTINFO program. In the structure incorporating 167Y, the hydroxyl (OH) group of the tyrosine makes a hydrogen bond with the nitrogen atom of glycine 108 (G108) (Figure S4). This hydrogen bond is not present with F167, or with the other amino acids commonly observed at this position.

#### Surrogate migration assay using CCR3

As an indirect test of possible function of CCRL2-F167Y, HEK293 cells expressing human CCR3 containing either phenylalanine (^169^F) or tyrosine (^169^Y) at the homologous position were tested for migration in a chemotaxis assay. Mutation of ^169^F to ^169^Y in CCR3 markedly reduced (3-fold) the migration of HEK293 cells in response to eotaxin (Figure S5), indicating a role of ^169^F in CCR3 function and by analogy suggesting that CCRL2-F167 may be a functionally superior receptor than CCRL2-167Y.

#### Chemotaxis of CCRL2

We tested chemotactic responses of HEK293 cells transfected with either CCRL2-F167 or CCRL2-167Y. We observed no chemotactic responses to a panel of 13 chemokines or peptides including the HIV-1 inhibitory chemokines CCRL3, 4, 5 and CXCL12, suggesting CCRL2 is not a receptor of these chemokines. However, our experiments did not include the recently identified CCRL2 ligands, chemerin and CCL19 [51], [52], due to availability.

## Discussion

We investigated the impact of genetic variation of 3p21–22 chemokine receptor genes on HIV/AIDS in this population-based association study. We identified a total of 6 exonic variants in *CCR3*, *CCR8*, *CXCR6* and *CCRL2* in 144 samples with extreme phenotypes. Through Cox regression survival analysis of the variants in HIV-1 natural cohorts, we identified three polymorphisms (*CCRL*2-Y167F, *CCR*3-255C and *CCR8*-*27G*) as having previously unidentified correlation with AIDS progression that appear to confer additional effect beyond the well-studied AIDS-modifying polymorphisms *CCR5*-Δ32, *CCR5*-P1/*CCR5*-2459A, and *CCR2*-64I. *CCRL2*-167F was associated with strong accelerated progression to AIDS, resulted almost entirely from rapid development of the AIDS-defining disease PCP.

The coverage of SNPs in the 3p21–22 region is limited in the GWAS SNP chips. Several exonic SNPs (*CCRL2*-167F, -243V, *CCR8*-27G, *CCR5*-2459A, *CCR5*- Δ32) genotyped in this study were not included in the SNP chips that have been used in GWAS for HIV-1 [3], [5]–[8], highlighting the ongoing need of candidate gene analysis in the GWAS era. The newly observed effects of chemokine coreceptor genes thus await further replication.

It must be cautioned that the combination of the presence of multiple AIDS associations in this chemokine receptor complex, and the LD between the receptor genes, makes determining the true source of the associations difficult. Overall, however, the association analysis points to receptor gene variant associations beyond those that can be attributed to the known AIDS affecting receptor gene polymorphisms. The association of additional chemokine receptors, beyond the primary CCR5 and CXCR4, with AIDS progression is plausible as several of these have been shown to bind to HIV in varying degrees [26], [35], [36], [39]–[42], [53]. The complexity of associations in this region makes it essential to identify a functional effect of the genetic variants on disease, before concluding that the associations are real. It should be noted that we may have not detected all existing SNPs in the region and was also underpowered in detecting rare SNPs (1%). We found that another *CCRL2* exonic SNP in the public domain (168M, rs6441977) was not associated with progression to AIDS-87 (RH = 0.84, 95% 0.50–1.39) in our seroconverters. We performed haplotype analysis for the three exonic SNPs in CCRL2 and found that only the haplotypes bearing rs3204849 A (*CCRL2* Y167F) and rs3204850 (V243I) were associated with AIDS; the haplotype bearing rs6441977A (V168M) had no effect. No additional information was gained by performing a haplotype analysis compared to single SNP analysis.

With these caveats we argue that the association of the *CCRL2*-167F variant is worthy of interest. First, within the noted limits of the association analysis, the association is quite strong (RH = 1.9, *P* = 0.002, corrected), remains significant with *CCR5*-Δ32 taken into account, and in an automatic selection analysis with all known factors taken into account. Second, although there is no direct demonstration of function for this polymorphism, and the F to Y substitution is generally a conservative one, several lines of phylogenetic, chemical modeling, and indirect experimental data suggest that CCRL2-167Y significantly alters the properties of this receptor.

Protein structure modeling data suggest that the risk-associated F to Y substitution could change the boundary of the transmembrane domain, and introduce a hydrogen bond. Of particular interest is the alignment data of the conservation of this residue among CC chemokine receptors. Strikingly, phenylalanine, the ancestral alternate variant to the AIDS risk tyrosine variant, or another nonpolar amino acid, occurs at this position in all receptors known to be functional [54]; a polar amino acid only occurs in the two cases of the nonsignaling DARC receptor. Further, substitution of tyrosine for phenylalanine at this position in the functional receptor CCR3 reduced migration of HEK293 cells in response to eotaxin threefold. We emphasize that all of these tests are *in silico* or indirect, and direct test of the effect of the F to Y substitution on the function of CCRL2 remains to be done; new knowledge of the functions and ligands of CCRL2 should make this more straightforward.

The association of CCRL2 with AIDS and PCP is unique as CCRL2 has not been shown to serve as a coreceptor for HIV-1. Our chemotaxis assay experiments excluded 11 common chemokines as ligands of CCRL2. The association does not appear to be a general function of increased HIV susceptibility, but instead specifically attributable to an increase in PCP among individuals carrying this receptor variant. PCP, caused by *Pneumocystis jirovecii*, is the most common opportunistic infection in untreated HIV-1-infected immunosuppressed persons. PCP is mediated by marked inflammatory responses in lung involving macrophages and chemokines and cytokines [55]. The association of CCL2 with PCP might be attributable to two aspects of CCRL2: immune regulation or direct interaction with HIV. CCRL2 may affect PCP through its immune regulating role at local inflammation sites, possibly by concentrating and presenting chemokines [51], [52], [56]. CCRL2 was rapidly upregulated in murine lung macrophages following inflammation induction [57] and deficiency of CCRL2 impaired lung dendritic cell migration [58]. Alternatively, CCRL2 may influence HIV coreceptors entry through interacting or sequestering with CCL5 or anti-HIV chemokines [56], or may serve as a coreceptor for some strains of HIV-1. The mechanism of *CCRL2*-F167Y effect on PCP remains to be explored.

In summary, this comprehensive study of the chromosome 3 chemokine receptor cluster region identified multiple genetic variants that associated with HIV disease. The strongest new association appears to result from an increased susceptibility to PCP, rather than from a specific effect on HIV. Added to the existing knowledge of the effect of the chromosome 3 chemokine receptors on HIV disease, which has been already been exploited therapeutically, our results affirm this gene complex as a fertile ground for further research, both for HIV and potentially for a broad range of additional diseases.

## Materials and Methods

### Ethics statement

Institutional review boards (IRB) at National Cancer Institute, National Institutes of Health and participating institutes approved the study protocols. Written informed consent was obtained from all study participants and/or their legal guardians.

### Study participants

The study group includes 674 HIV-1 seroconverter European Americans, 669 seronegatives enrolled in the following natural history HIV-1 cohort studies: Multicenter AIDS Cohort Study (MACS) [59], the San Francisco City Clinic Cohort Study (SFCCC) [60], AIDS Link to the Intravenous Experience (ALIVE) [61], Hemophilia Growth and Development Study (HGDS) [62], and the Multicenter Hemophilia Cohort Study (MHCS) [63]. Seroconversion date was estimated as the midpoint between the last seronegative and the first seropositive HIV-1 antibody test date (mean interval 0.79 years, range 0.07–3.0 years). The censoring date was the earliest of the date of the last recorded visit, or July 31, 1997 for the ALIVE cohort, or December 31, 1995 for all other cohorts, to avoid the confounding effect of highly active anti-retroviral therapy (HAART). A later censoring date was used for ALIVE cohort because few ALIVE participants received HAART prior to July 31, 1997 [64].

### Resequencing and SNP identification

A panel of 72 EA and 72 AA samples representing extreme phenotypes for infection and progression (rapid progression, long term non-progression, and infection resistance) were used for SNP identification. The 5′ and 3′ untranslated (UTR) and coding regions of the *CCR3*, *CCR8*, *CCRL2*, and *CXCR6* genes were PCR amplified by overlapping primer sets (Table S2). PCR products were resequenced by BigDye terminator (Applied Biosystems, Foster City, CA). We did not sequence or genotyping SNPs in *CCR1*, *CCXCR1*, *CCR9* and *CCR4* as they are not recognized as HIV-1 coreceptors [26], [33]–[36] (reviewed by [11], [37]).

### Genotyping of genetic variants

Genotyping was done using PCR-restriction fragment length polymorphism (RFLP) or TaqMan assays. PCR primer sequences, TaqMan probes and primers, PCR conditions, and restriction enzymes used to genotype each variant are listed in Table S1. Briefly, PCR was carried out with 35 cycles of denaturing at 94°C for 30 s, annealing at 54–60°C for 30 s and extension at 72°C for 45 s. TaqMan assays were performed according to the manufacturer's manual (Applied Biosystems, Foster City, CA). The *CX3CR1* variants V249I and T280M were typed as previously reported [65].

### Statistical analysis

Kaplan-Meier survival statistics and the Cox proportional hazards (PH) model (Cox PH model) were used to assess the effects of genetic variants on the time of progression from HIV-1 infection to AIDS (1987 CDC definition) [66], using PROC PHREG and LIFETEST of SAS version 9.13 (SAS, Cary, North Carolina). For SNPs that showed significant association with AIDS development, explanatory analyses were performed for their specific impact on the AIDS-defining diseases Pneumocystis pneumonia (PCP), Kaposi's sarcoma, microbacterial avium infection, and cytomegalovirus. The relative hazard (RH) and significance of associations were determined using a Cox PH model without or with adjustment for confounding genetic factors not on chromosome 3: for EA HLA-B*27 and HLA-B*57, HLA-B*35Px, and *HLA* Class I homozygosity [1], [67], [68]; for AA *CCRL5*-In1.1, HLA-B*57 and *HLA* Class I homozygosity [1], [19], [67]. *CCR2-64I*, *CCR5*-Δ32 and *CCR5*-2459 were also included as covariates in the adjusted multivariable regression analysis. *CCR5* promoter haplotypes (P1, P2, and P4) are tagged by SNPs *CCR5*-2459 (rs1799987) and rs2734648 [43]. A visual inspection of the data with Kaplan-Meier survival curves was performed to determine the genetic models to be used in the Cox PH regression model. A dominant genetic model was tested for all genetic factors in this study, except for *CCR5*-2459 (recessive) [15], [16]. Participants were stratified by sex and by age at seroconversion: 0–20, >20–40, and over 40 years.

To determine the best explanatory set of genetic variants, while minimizing the number of comparisons in model selection, We used StepAIC procedure to build Cox proportional hazards models for the AIDS-1987 phenotype based on stepwise regression, Akaike information criteria (AIC), and the best subset selection [69]. Here we used PROC PHREG in SAS software with SLENTRY = 0.99 and SLSTAY = 0.995 (values chosen close to 1 to generate a sequence of models from null models to full model ordered by AIC) [48]. Model uncertainty caused by including large number of variables can be estimated by shrinkage (LR-p)/LR, where *LR* denotes the likelihood ratio *χ* ^2^ and *p* denotes the number of the predictors in the final model. A shrinkage below 0.85 raises concern of overfitting [48]. It is recommended that no more than m (the number of uncensored event)/10 predictor degree of freedom p (number of parameters) should be examined to fit a multiple regression model. As in our sample, there were 194 events without censoring, we expect that fitting with <19 variables would be appropriate [48].

To control for potential population stratification, we adjusted the regression analysis with eigenvector values [70]. Eigenvector values were obtained by performing principal component analysis of 700,022 SNPs from a previous GWAS study carried out in the same samples [71]. In the Cox regression analysis, eigenvector values for the top two principle components were included as covariates. The genomic inflation factor in this seroconverter population showed minimal systematic overall bias due to population structure in regards to disease progression phenotype as it was quite close to 1.0 (λ = 1.01) (expected under no population stratification) [71].

We further corrected for multiple comparisons by counting 7 chemokine receptor variants tested using a Bonferroni step-down (Holm) correction method [47], as implemented in the MULTITEST procedure in SAS.

The “generalized” R^2^ statistic in Cox model is based on the likelihood-ratio statistic (LRT) for testing the global null hypothesis [72]. The formula is given as: R^2^ = 1−e^−(LRT/n)^, where LRT = −2logL(0)−[−2logL(p)], n is the total sample size, logL(0) is the log-likelihood for a null model with no covariates, and logL(p) is the log-likelihood for the fitted model with p covariates.

We quantified LD between all pairs of biallelic SNPs using the absolute unsigned value of Lewontin's D′ statistic [73]. P values represent significance of departure from the null hypothesis that the pair is in equilibrium. All *P* values are two-tailed. Haploview was used for the LD plots.

### Prediction of CCRL2 protein structures

Three-dimensional models of the two CCRL2 proteins with 167F or 167Y were constructed using the PROTINFO structure prediction server (http://www.protinfo.compbio.washington.edu), using the comparative modeling protocol [74], [75]. The detailed modeling method is presented in Text S1. The CCRL2 TM topology was established using ConPred II (http://bioinfo.si.hirosaki-u.ac.jp/~ConPred2/), a predication program based on consensus results of several prediction methods including TMpred, TMAP,TMHMM, HMTOP and MEMSAT [50].

### Expression of human CCRL2 in HEK293 cells

cDNAs coding for the full-length open reading frame (ORF) of human CCRL2 carrying 167Y or 167F were PCR amplified from two individuals with the respective homozygous genotype with proofreading DNA polymerase pfu (Stratage, La Jolla, CA). After confirmation of sequence accuracy, they were ligated into the pcDNA3 (Invitrogen, Gaithersburg, MD). Human embryonic epithelial cells line HEK293 was transfected with the constructs of CCRL2-167F or CCRL2-167Y. Stable transfectants were selected by culturing the cells in 800 µg/ml G418. Once the stable cell lines were established, they were examined for chemotactic responses to chemokines.

### Chemotaxis assays

Migration of CCRL2-167F and CCRL2-167Y transfected HEK293 cells was assessed using a 48-well microchemotaxis chamber technique. They were examined for chemotactic responses to the following chemokines: CCL5, CCL3, CCL4, CCL2, CCL8, CCL7, CCL11, CXCL12, CCL21, CCL20, and CXCL10. The cells were also tested for migration in response to chemotactic peptides using formayl peptide receptors, including W peptide and MMK-1 (Text S1).

### Expression of human CCR3 in HEK293 cells

A cDNA coding for ^169^Y-CCR3 was created from the wild-type human ^169^F-CCR3 ligated into the pcDNA3, which were used to transfect HEK293 cells (Text S1).

## Supporting Information

Figure S1 Linkage disequilibrium of variants in 3p21 chemokine receptor genes. LD was shown for D′ and r^2^ in European Americans.
(TIF)

Figure S2 Alignment of a segment of sequences corresponding to the CCRL2-F167Y residue from chemokine receptor genes.
(TIF)

Figure S3 Predicted CCRL2 transmembrane topology change by F167Y change.
(TIF)

Figure S4 Three-dimensional models of the 167Y (top) and F167 (bottom) CCRL2 proteins. The models are shown with virtual bonds connecting the CA atoms. The atoms in residue 167 are shown in spacefill view, with the carbons in white, oxygens in red and nitrogens in blue. The hydroxyl (OH) group of 167Y forms a hydrogen bond with the nitrogen atom of glycine 108, which is not present in the F167 allele.
(TIF)

Figure S5 Migration of HEK293 cells expressing wild-type (^169^F) or mutated (^169^Y) CCR3 in response to eotaxin. A. Surface expression of CCR3 on each cell line was evaluated by flow cytometry with anti-CCR3 mouse monoclonal Ab. B. Migration of each cell line in response to 1, 10 or 100 ng/ml of eotaxin was evaluated and presented as chemotactic index (CI).
(TIF)

Table S1 Genotyping primers, PCR conditions, and Restriction Enzymes.
(DOC)

Table S2 Primer sequences for PCR amplification and direct sequencing.
(DOCX)

Text S1 Supplementary methods.
(DOC)
