# Supplementary figures and images for: Role of Exonic Variation in Chemokine Receptor Genes on AIDS: CCRL2 F167Y Association with Pneumocystis Pneumonia
Source: PLoS Genet. 2011 Oct 27;7(10):e1002328. doi: 10.1371/journal.pgen.1002328 (PMC3203199; doi:10.1371/journal.pgen.1002328)

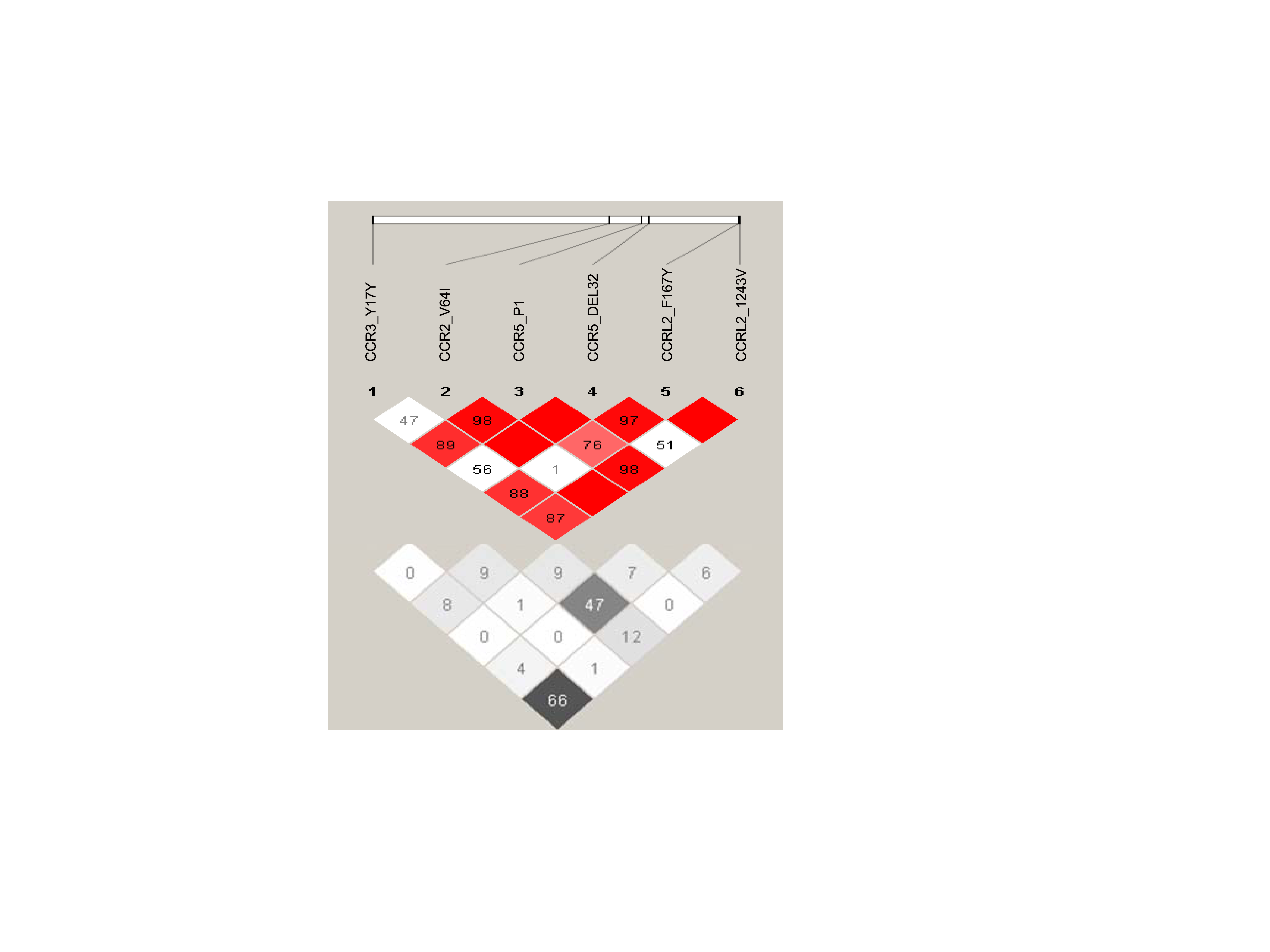

Supplement: Figure S1 — Linkage disequilibrium of variants in 3p21 chemokine receptor genes. LD was shown for D′ and r2 in European Americans. (TIF) [file pgen.1002328.s001.tif]

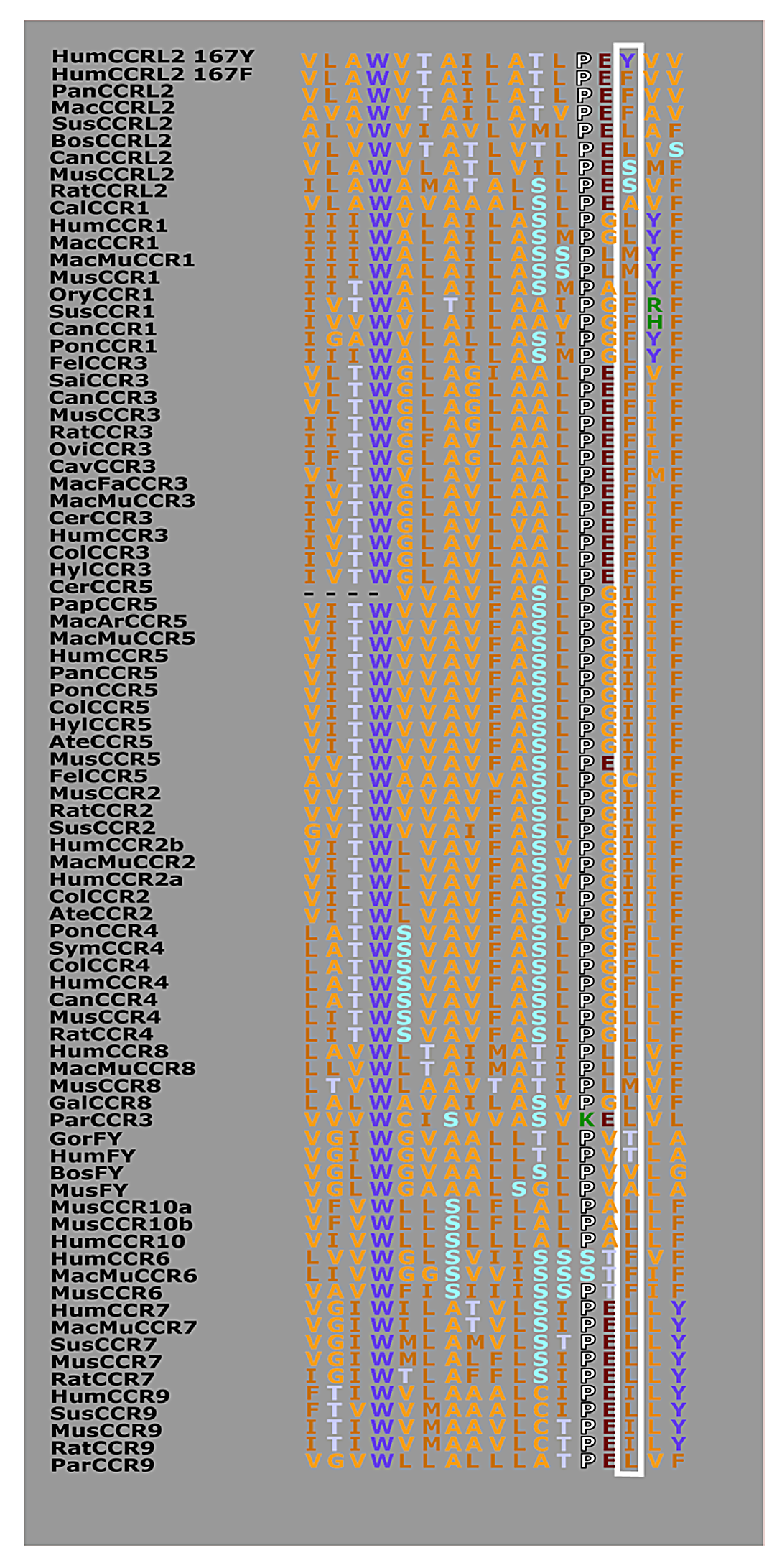

Supplement: Figure S2 — Alignment of a segment of sequences corresponding to the CCRL2-F167Y residue from chemokine receptor genes. (TIF) [file pgen.1002328.s002.tif]

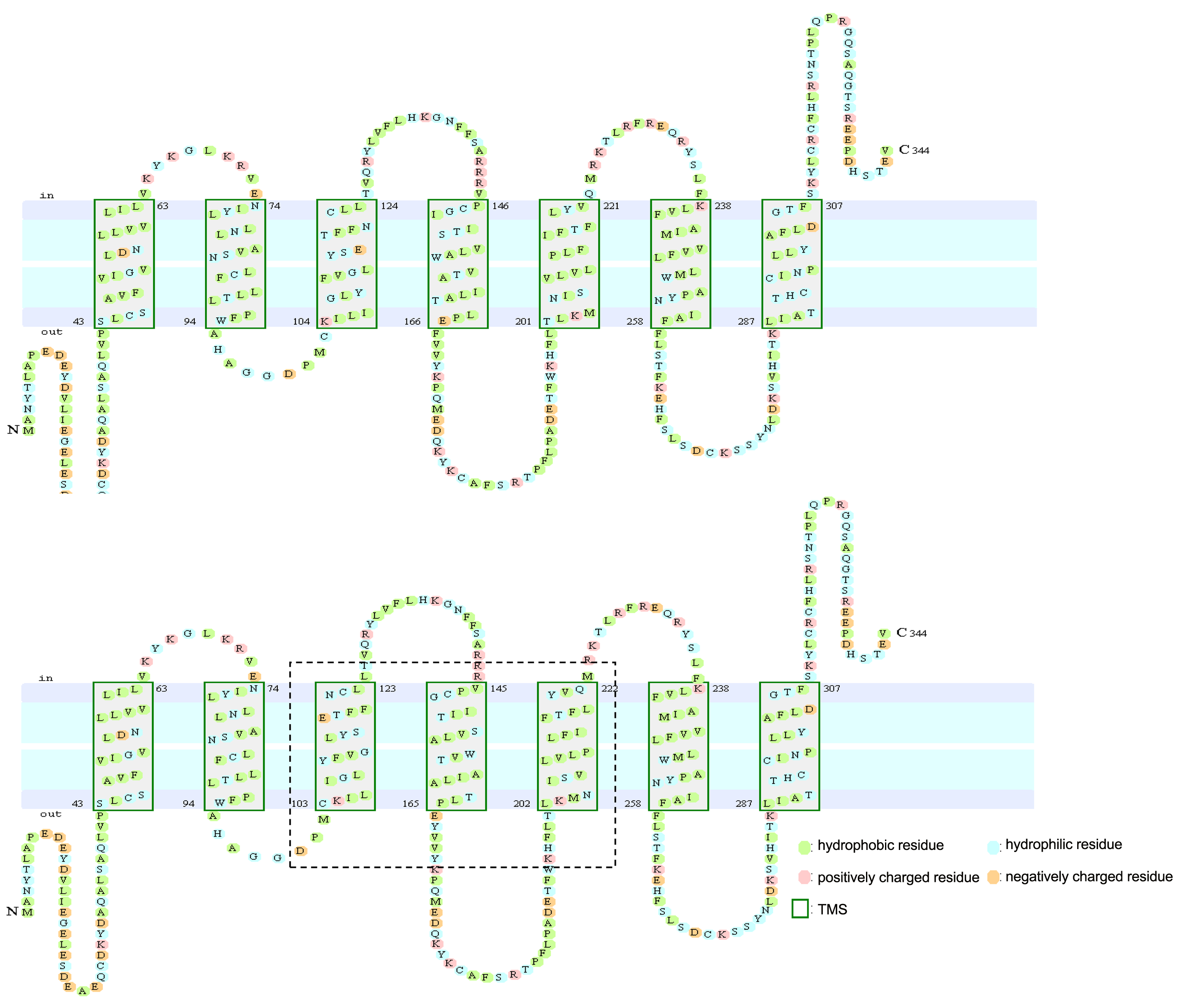

Supplement: Figure S3 — Predicted CCRL2 transmembrane topology change by F167Y change. (TIF) [file pgen.1002328.s003.tif]

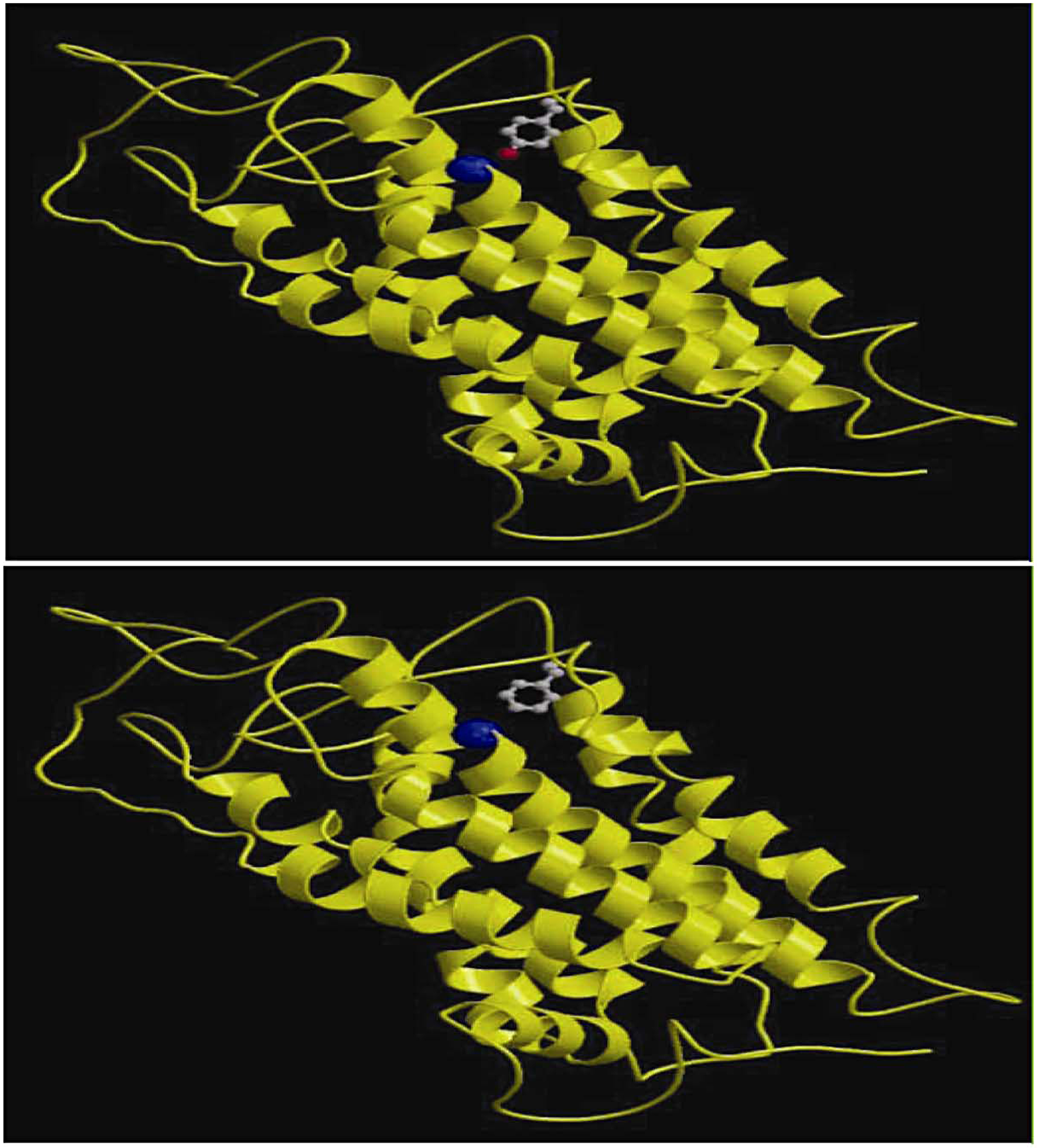

Supplement: Figure S4 — Three-dimensional models of the 167Y (top) and F167 (bottom) CCRL2 proteins. The models are shown with virtual bonds connecting the CA atoms. The atoms in residue 167 are shown in spacefill view, with the carbons in white, oxygens in red and nitrogens in blue. The hydroxyl (OH) group of 167Y forms a hydrogen bond with the nitrogen atom of glycine 108, which is not present in the F167 allele. (TIF) [file pgen.1002328.s004.tif]

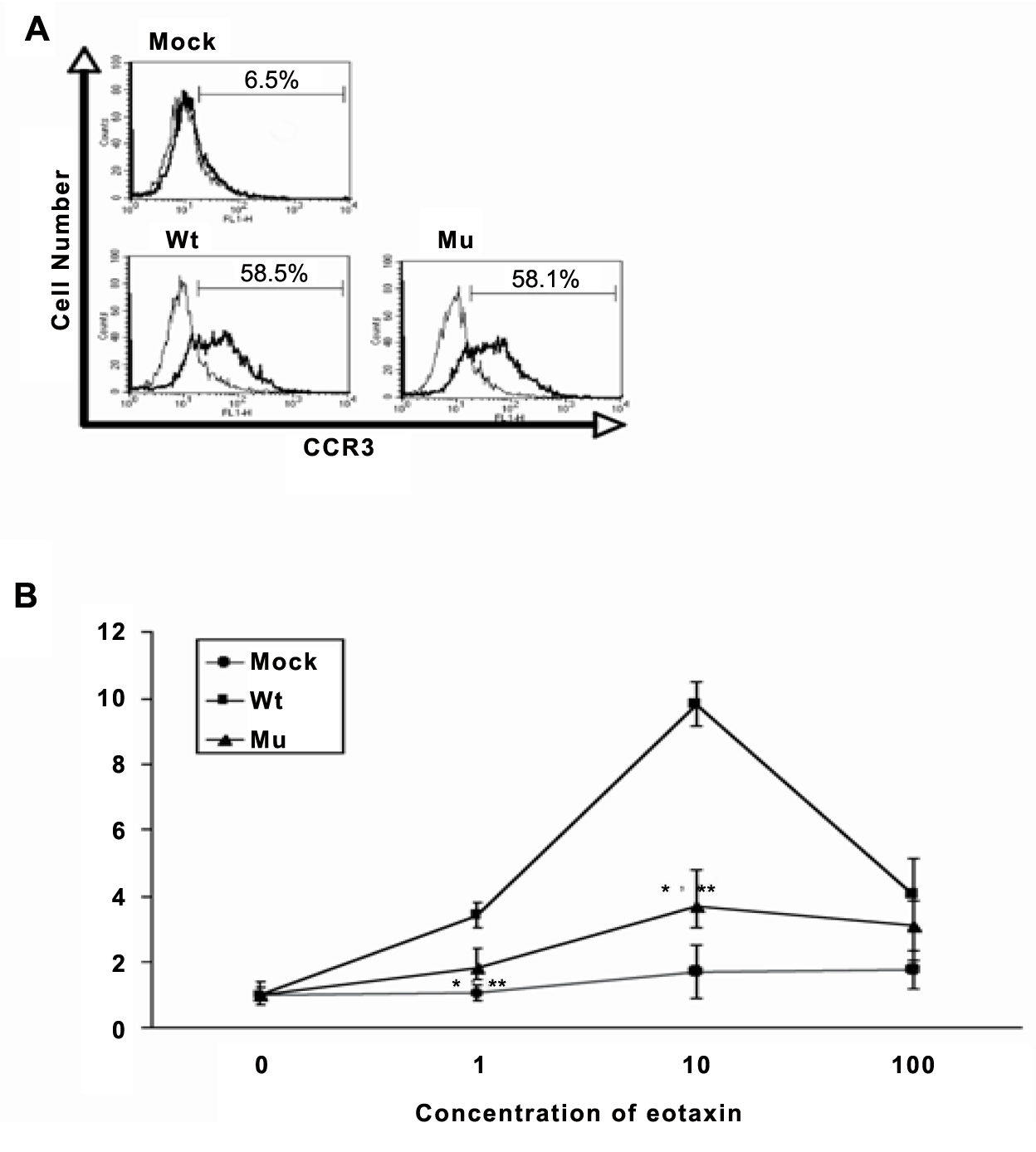

Supplement: Figure S5 — Migration of HEK293 cells expressing wild-type (169F) or mutated (169Y) CCR3 in response to eotaxin. A. Surface expression of CCR3 on each cell line was evaluated by flow cytometry with anti-CCR3 mouse monoclonal Ab. B. Migration of each cell line in response to 1, 10 or 100 ng/ml of eotaxin was evaluated and presented as chemotactic index (CI). (TIF) [file pgen.1002328.s005.tif]
