# Supplementary material for: Role of Exonic Variation in Chemokine Receptor Genes on AIDS: CCRL2 F167Y Association with Pneumocystis Pneumonia
Source: PLoS Genet. 2011 Oct 27;7(10):e1002328. doi: 10.1371/journal.pgen.1002328 (PMC3203199; doi:10.1371/journal.pgen.1002328)
Supplement: Table S1 — Genotyping primers, PCR conditions, and Restriction Enzymes. (DOC) [file pgen.1002328.s006.doc]

Table S1. Genotyping primers, PCR conditions and restriction enzymes

| Variant | Method | Forward primer | Reverse primer | Tann (ºC) | Mg++ | Enzyme |
| --- | --- | --- | --- | --- | --- | --- |
| *CCR8-*27G | MS-PCR^1^ | TCTCAAGCCCCTGTGA**T**GG | GGGCCAGGTTCAAGAGGTAT | 60 | 1.5 |  |
|  |  | CCGACTACTACTACCCTGATATCT**AA**TCAAGCCCCTGTGA**T**GC | |  |  |  |
| *CCR3*-255C | TaqMan | GGAGAAGTGAAATGACAACCTCAC | AGTGCTCTGGTATCAGCTTTTTCA |  |  |  |
|  |  | FAM-CATCCTACTATGATGACGTG | VIC-ATCCTACTAcGATGACGTG |  |  |  |
| *CCR3*-P39L | RFLP | CTATCACAGGGAGAAGTGAAATGA | ACCCTGAGAGGAGCTTACACA | 59 | 1.5 | MspA1I |
| *CXCR6*-E3K | Forced-RFLP^2^ | CTATTTGCCCCCTAAATGT | CATAGTCTTCATGGTAATCAAGC | 54 | 2 | HindIII |
| *CCRL2*-Y167F | RFLP | GGCGATCCCATGTGTAAAAT | CAGAAAATGCTTCCAGAATGTC | 60 | 2 | EcoRI |
| CCRL2-V168M | RFLP | CCCTGTGGCATCATTACAAG | TGCTTCCAGAATGTCTCATCA | 60 | 1.5 | NlaIII |
| *CCRL2*-I243V | Forced-RFLP^2^ | TGAACATTTCGGTTCTTGTCC | CACATCAGAAGGAAGACTACCAGTA | 57 | 1.5 | RsaI |

^1^In mutagenically separated PCR (MS-PCR), two forward primers were used; underlined nucleotide was artificially changed to enhance detection specificity;

^2^Underlined nucleotide was artificially changed to create a restriction site.
