# Supplementary material for: Role of Exonic Variation in Chemokine Receptor Genes on AIDS: CCRL2 F167Y Association with Pneumocystis Pneumonia
Source: PLoS Genet. 2011 Oct 27;7(10):e1002328. doi: 10.1371/journal.pgen.1002328 (PMC3203199; doi:10.1371/journal.pgen.1002328)
Supplement: Table S2 — Primer sequences for PCR amplification and direct sequencing. (DOCX) [file pgen.1002328.s007.docx]

**Table S2. Primer sequences for PCR amplification and direct sequencing**

| Primer pair | Forward (5’->3’) | Reverse (5’->3’) |
| --- | --- | --- |
| CCR3-1 | AAGCTTCAGCTCTTTCCTTCC | CAGGCCCACGTCATCATAGT |
| CCR3-2 | AGGGAGAAGTGAAATGACAACC | CAGAATGGAAGGGTGACGAG |
| CCR3-3 | AACCTGGCCATTTCGGAC | GACGATGCTGGTGATGACAC |
| CCR3-4 | CATGCTGTGTTTGCCCTTC | AGCAGCGTTTTGATGATTCC |
| CCR3-5 | TCTCCCTCTGCTCGTTATGG | GAACCTCTCTCCAACAAAGGC |
| CCR3-6 | CGCCTACTCCCACTGCTG | ATTTTCTGCATCTGACCTAAAACA |
| CCR3-7 | CAGAGCCGGAACTCTCTATTG | CTGCTTTTTAGGGTTGATGATG |
| CCR3-8 | TGGGCAGCGTACTCATCA | AGCTTTTCATTAAGCAGGGAAA |
| CCR8-1 | CTTTTATGTGTCTCTGTGACCAGG | GATGCTCCTCAGCTTCTTGC |
| CCR8-2 | CTGGTCATCCTGGTCCTTGT | CTTTAGGGCATACACGGCAT |
| CCR8-3 | AGCAGCATGTTTTTCATCACC | GTGAATGGGATCAACAAGCC |
| CCR8-4 | GATCTTCACCAACTTCAAAATGA | GTGACATGGGTGGCATAAGT |
| CCR8-5 | ATCTTGGATGGATGTAGCATAAG | TTTAGTCTTCATTGATCCTCACAA |
| CCRL2-1 | ATGGCCAATTACACGCTG | TTACACTTCGGTGGAATGGTC |
| CXCR6-1 | ATCCTCAGCCCCAAATATAA | TATGACCAGCACCAGAGAGT |
| CXCR6-2 | GTGGTGTTTGTCTGTGGTCT | AGCATGGACGTGTAGAAGTTA |
| CXCR6-3 | ATGGGTGTTTGGCCAGGT | GGGCAAGGAAACCAGCAG |
| CXCR6-4 | CCAGCTTGCTCATCTGGGT | GAAGCCTCCAGCATGAAGC |
| CXCR6-5 | CCATGATTGTCTGCTATTCAG | CATAGAGCACAGGGTTAAGG |
| CXCR6-6 | CAGAGGCCATCGCATACCT | AGTTTTTCGAAACCCTGGCA |
