# Supplementary material for: Role of Exonic Variation in Chemokine Receptor Genes on AIDS: CCRL2 F167Y Association with Pneumocystis Pneumonia
Source: PLoS Genet. 2011 Oct 27;7(10):e1002328. doi: 10.1371/journal.pgen.1002328 (PMC3203199; doi:10.1371/journal.pgen.1002328)
Supplement: Text S1 — Supplementary methods. (DOC) [file pgen.1002328.s008.doc]

**Text S1.** **Supplementary methods.**

**Prediction of CCRL2 protein structures**

Three-dimensional models of the two CCRL2 alleles were constructed using the PROTINFO structure prediction server (http://www.protinfo.compbio.washington.edu). Modeling was performed using the comparative modeling protocol [1,2]. The structure of bovine rhodopsin (the only 7TM receptor for which three-dimensional structures have been solved, PDB identifier 1gzm-A) was determined to be the closest structural homologue and was used as template to construct the initial models of the CCRL2 sequences. Initial models were constructed using a minimum perturbation approach that aims to preserve as much information as possible from the template structure solved by x-ray crystallography. Variable side chains and main chains (in the CCRL2 protein relative to the template sequence) were then built using a graph-theory clique-finding approach that explores a variety of possible conformations for the respective side chains and main chains and finds the optimal combination using an all-atom scoring function [3-5].

**Expression of human CCR3 in HEK293 cells.**

A cDNA coding for ^169^Y-CCR3 was created from the wild-type human ^169^F-CCR3 ligated into the pcDNA3 (Invitrogen, Gaithersburg, MD) (a kind gift from Dr. Takeo Edamatsu) by site-directed mutagenesis using the QuickChange II Kit (Stratagene, La Jolla, CA). The pcDNA3 constructs carrying the wild-type (^169^F) or mutated (^169^Y) CCR3 cDNA were used to transfect HEK293 cells. The transfected cells were cultured in DMEM (Cambrex, Walkersville, MD) containing 10% FCS for 2 weeks in the presence of 1.5 mg/ml G418 (Invitrogen, Gaithersburg, MD) before use. Expression of cell surface CCR3 was evaluated by flow-cytometry analysis. A half million cells were suspended in 50 µl of cold PBS containing 0.02% sodium azide, 1% FCS and 20 µg/ml of human IgG, incubated for 15 min at room temperature, and with FITC-conjugated anti-human CCR3 mouse monoclonal antibody (R&D Systems, Minneapolis, MN) for 15 min at room temperature. At the end of the incubation, 7AAD was added to each tube (100 µM). The cells were washed with PBS, and subsequently analyzed by flow cytometry using a FACScan (Becton Dickinson).

**Chemotaxis assays**

migration of CCRL2-167F and CCRL2-167Y transfected HEK293 cells was assessed using a 48-well microchemotaxis chamber technique. They were examined for chemotactic responses to the following chemokines: CCL5, CCL3, CCL4, CCL2, CCL8, CCL7, CCL11, CXCL12, CCL21, CCL20, and CXCL10. The cells were also tested for migration in response to chemotactic peptides using formaylpeptide receptors, including W peptide and MMK-1. Different concentrations of chemokines (PeproTech) were placed in wells of the lower compartment of the chamber (Neuro Probe, Cabin John, MA), the cell suspension (50 μl of 1 X 10^6^/ml) was seeded into wells of the upper compartment which was separated from the lower compartment by a polycarbonate filter (Osmonics, Livermore, CA; 5 μm-pore size for leukocytes, 10 μm pore-size). The filters were precoated with 50 μg/ml collagen type I (Collaborative Biomedical products, Bedford, MA) to favor cell attachment. After incubation at 37oC for 300 min, the filters were removed, stained and the number of cells migrating across the filters was counted by light microscopy. The experiments were performed 3 times with each cell type and the results are presented as the chemotaxis indexes (CI) representing the fold increase in the number of migrating cells in response to stimuli, over the spontaneous cell migration (in response to control medium). Student’s *t* test was used to analyze the differences in cell migration in response to stimulants versus control medium.

**References**

1. Hung LH, Ngan SC, Liu T, Samudrala R (2005) PROTINFO: new algorithms for enhanced protein structure predictions. Nucleic Acids Res 33: W77-80.

2. Hung LH, Samudrala R (2003) PROTINFO: Secondary and tertiary protein structure prediction. Nucleic Acids Res 31: 3296-3299.

3. Samudrala R, Moult J (1998) An all-atom distance-dependent conditional probability discriminatory function for protein structure prediction. J Mol Biol 275: 895-916.

4. Samudrala R, Levitt M (2002) A comprehensive analysis of 40 blind protein structure predictions. BMC Struct Biol 2: 3.

5. Samudrala R, Moult J (1998) A graph-theoretic algorithm for comparative modeling of protein structure. J Mol Biol 279: 287-302.
